# Adsorption of Cu(II) and Ni(II) from Aqueous Solutions Using Synthesized Alkali-Activated Foamed Zeolite Adsorbent: Isotherm, Kinetic, and Regeneration Study

**DOI:** 10.3390/molecules29102357

**Published:** 2024-05-16

**Authors:** Eliška Svobodová, Zdeněk Tišler, Kateřina Peroutková, Kateřina Strejcová, Jan Abrham, Josef Šimek, Zahra Gholami, Mohammadtaghi Vakili

**Affiliations:** 1 ORLEN UniCRE, a.s., Revoluční 1521/84, 400 01 Ústí nad Labem, Czech Republic; eliska.svobodova@orlenunicre.cz (E.S.); zdenek.tisler@orlenunicre.cz (Z.T.); katerina.peroutkova@orlenunicre.cz (K.P.); katerina.strejcova@orlenunicre.cz (K.S.); abrhamj@vscht.cz (J.A.); zahra.gholami@orlenunicre.cz (Z.G.); 2 Faculty of Science, Jan Evangelista Purkyně University in Ústí nad Labem, Pasteurova 3632/15, 400 96 Ústí nad Labem, Czech Republic; josef.simek@ujep.cz

**Keywords:** adsorption, heavy metals, zeolite foam, alkali activation

## Abstract

Water pollution, particularly from heavy metals, poses a significant threat to global health, necessitating efficient and environmentally friendly removal methods. This study introduces novel zeolite-based adsorbents, specifically alkali-activated foamed zeolite (AAFZ), for the effective adsorption of Cu(II) and Ni(II) ions from aqueous solutions. The adsorbents’ capabilities were comprehensively characterized through kinetic and isotherm analyses. Alkaline activation induced changes in chemical composition and crystalline structure, as observed via XRF and XRD analyses. AAFZ exhibited a significantly larger pore volume (1.29 times), higher Si/Al ratio (1.15 times), and lower crystallinity compared to ZZ50, thus demonstrating substantially higher adsorption capacity for Cu(II) and Ni(II) compared to ZZ50. The maximum monolayer adsorption capacities of ZZ50 and AAFZ for Cu(II) were determined to be 69.28 mg/g and 99.54 mg/g, respectively. In the case of Ni(II), the maximum monolayer adsorption capacities for ZZ50 and AAFZ were observed at 48.53 mg/g and 88.99 mg/g, respectively. For both adsorbents, the optimum pH for adsorption of Cu(II) and Ni(II) was found to be 5 and 6, respectively. Equilibrium was reached around 120 min, and the pseudo-second-order kinetics accurately depicted the chemisorption process. The Langmuir isotherm model effectively described monolayer adsorption for both adsorbents. Furthermore, the regeneration experiment demonstrated that AAFZ could be regenerated for a minimum of two cycles using hydrochloric acid (HCl). These findings highlight the potential of the developed adsorbents as promising tools for effective and practical adsorption applications.

## 1. Introduction

The accessibility of clean water has become increasingly challenging in recent decades due to the rapid expansion of industrial activities and population growth. These developments have led to significant pollution and degradation of natural water reservoirs, thereby exacerbating the scarcity of uncontaminated potable water [1]. Heavy metals have emerged as a significant concern among the various pollutants contaminating water sources [2]. Primarily originating from industrial discharges, these contaminants are particularly problematic because, unlike organic pollutants, heavy metals are not biodegradable. Consequently, they tend to accumulate in ecosystems, posing severe health risks due to their potential to enter the human body through the food chain [3,4]. The toxicity of heavy metals is associated with a wide range of health issues, including but not limited to respiratory, renal, cerebral, reproductive, cardiac, and immunological disorders [5,6].

Amongst the heavy metals, Cu(II) and Ni(II) are prevalent toxic pollutants, commonly discharged from diverse industrial processes including refining and electroplating. Consequently, aqueous effluents from such industries often exhibit high concentrations of these metal ions, posing significant environmental and human health risks. Cu(II) contamination predominantly arises from smelting, electroplating, and mining activities, with excessive intake leading to severe health implications such as mortality, neurological damage, hypoglycemia, gastrointestinal distress, and cramps [7]. Ni(II) emissions stem mainly from electroplating facilities, thermal power plants, nickel-cadmium battery production, and metallurgical operations, contributing to chronic and acute health disorders including dermal reactions, hepatotoxicity, genotoxicity, nephrotoxicity, gastrointestinal disturbances, neurotoxicity, and carcinogenesis in humans [8].

Given these important consequences, there is a crucial need for effective methods to reduce or remove heavy metal contamination in water. Various approaches, such as chemical precipitation, ion exchange, electrolysis, oxidation, and adsorption, have been studied to address this issue [1]. Among these methods, adsorption emerges as particularly noteworthy due to its operational simplicity, efficiency, cost-effectiveness, minimal environmental impact, minimal energy requirements, absence of secondary pollution, and adaptability to diverse applications, thereby rendering it a favored approach for water remediation [9,10].

The effectiveness and success of the adsorption process heavily rely on the properties of the adsorbent material employed. Various types of adsorbent materials have been extensively studied for their capability to remove heavy metals, including residues from industrial and agricultural processes, metal–organic frameworks, activated carbon, mesoporous silica, clays, fly ash, and zeolites, as well as adsorbents derived from alternative sources.

Recent progress in environmental remediation has underscored the importance of developing cost-effective adsorbents specifically designed for extracting heavy metal pollutants from water. These adsorbents, mainly derived from inorganic sources, offer economic advantages and demonstrate superior properties compared to organic alternatives, such as mechanical strength, thermal stability, and chemical resistance [11]. Among the wide range of adsorbents investigated, clays and zeolites have received significant attention due to their affordability, abundance, diverse chemical compositions, and high adsorption capacities [1,12]. Zeolites, in particular, have been highly regarded for their versatile applications across various sectors for more than two centuries, highlighting their extensive history of utility.

Using natural zeolites in environmental remediation and water treatment exploits their inherent advantages, including cost-effectiveness, intricate microporous structure, ion exchange capacity, and notable thermal and chemical stability [13]. These microporous minerals are characterized by their unique assembly of alumina (Al_2_O_3_) and silica (SiO_2_) tetrahedra, forming a three-dimensional network. A notable characteristic of zeolites is the isomorphous substitution of Al^3+^ ions and Si^4+^, which imparts a negative charge to the surface. This negative charge necessitates the presence of counter-cations to maintain electrostatic equilibrium, thereby facilitating the exchange of these ions with other cations present in aqueous solutions [14,15].

The utilization of zeolites as adsorbents has garnered significant attention from the research community due to their remarkable adsorptive properties. These properties stem from a combination of ion exchange and molecular sieving capabilities, which can be further enhanced through structural modifications [12]. Improving the selectivity of zeolites for specific impurities often involves their integration with additional materials such as limestone or clays. However, achieving optimal performance often necessitates further modifications to the zeolite structure to enhance the clearance of the internal pore network and increase the availability of specific surface area and adsorption sites. Among the techniques utilized for this purpose, foaming methodologies, such as template-based foaming or alkaline activation of zeolites, have proven to be effective [16]. In particular, the alkaline activation process, which involves treating aluminosilicate precursors to induce foaming, enables the synthesis of inorganic foam structures at relatively low temperatures. This procedure generates hydrogen and oxygen gases under highly alkaline conditions, resulting in the formation of exceptionally porous materials [17].

To the best of our knowledge, the adsorptive potential of such foamed materials for separating heavy metals from aqueous solutions remains unexplored in existing literature. Addressing this gap, the present study aims to innovate by developing novel, economically viable materials based on zeolite through a dual-stage process of alkali activation followed by foaming. The resulting material, termed alkali-activated foamed zeolite (AAFZ), is studied for its capacity to remove heavy metal species, specifically Ni(II) and Cu(II), from water. This study meticulously assesses the efficacy of AAFZ through a series of batch adsorption experiments, alongside isotherm and kinetic analyses, complemented by various characterization techniques. Through this investigation, the main aim is to emphasize the originality and novelty of the work, highlighting the significant contributions of AAFZ to the realms of environmental science and water-purification technologies.

## 2. Results and Discussion

### 2.1. Characterization

The XRF analysis of ZZ50 and AAFZ samples (see Table 1) revealed notable differences in oxide parameters following modification. Post-treatment, AAFZ exhibited significant increases in Mg, K, and Na content compared to ZZ50. Specifically, Mg content increased from 0.78% in ZZ50 to 3.19% in AAFZ, while K content rose from 4.26% in ZZ50 to 8.63% in AAFZ. Similarly, Na content showed a marked elevation from 0.3% in ZZ50 to 5.12% in AAFZ. These changes, introduced via alkaline activators and MgO addition during synthesis, substantially enhanced the mechanical properties of the materials [18].

Moreover, AAFZ displayed a decrease in SiO_2_ and Al_2_O_3_ content compared to ZZ50, indicating a significant alteration in both SiO_2_ and Al_2_O_3_ composition. Specifically, SiO_2_ and Al_2_O_3_ contents decreased from 75.30% and 12.60% in ZZ50 to 68.80% and 10% in AAFZ. These compositional differences highlight the distinct chemical profiles among the samples, potentially affecting their effectiveness in heavy metal adsorption applications. Additionally, AAFZ showed a slight decrease in Si/Al ratios compared to ZZ50. The Si/Al ratio in AAFZ increased by 1.15 times, reaching 5.84. These variations are likely due to differences in synthesis methodologies, where the incorporation of alkaline activators and foaming agents exerted considerable influence on the Si/Al ratio, thereby impacting surface chemistry and ion exchange capacity. Furthermore, changes in chemical composition may have significant implications for material stability, porosity, and surface area, which are critical parameters governing adsorption efficiency. These findings underscore the essential role of synthesis techniques in tailoring the characteristics of adsorbent materials for optimal performance in environmental remediation applications.

**Figure 1:**
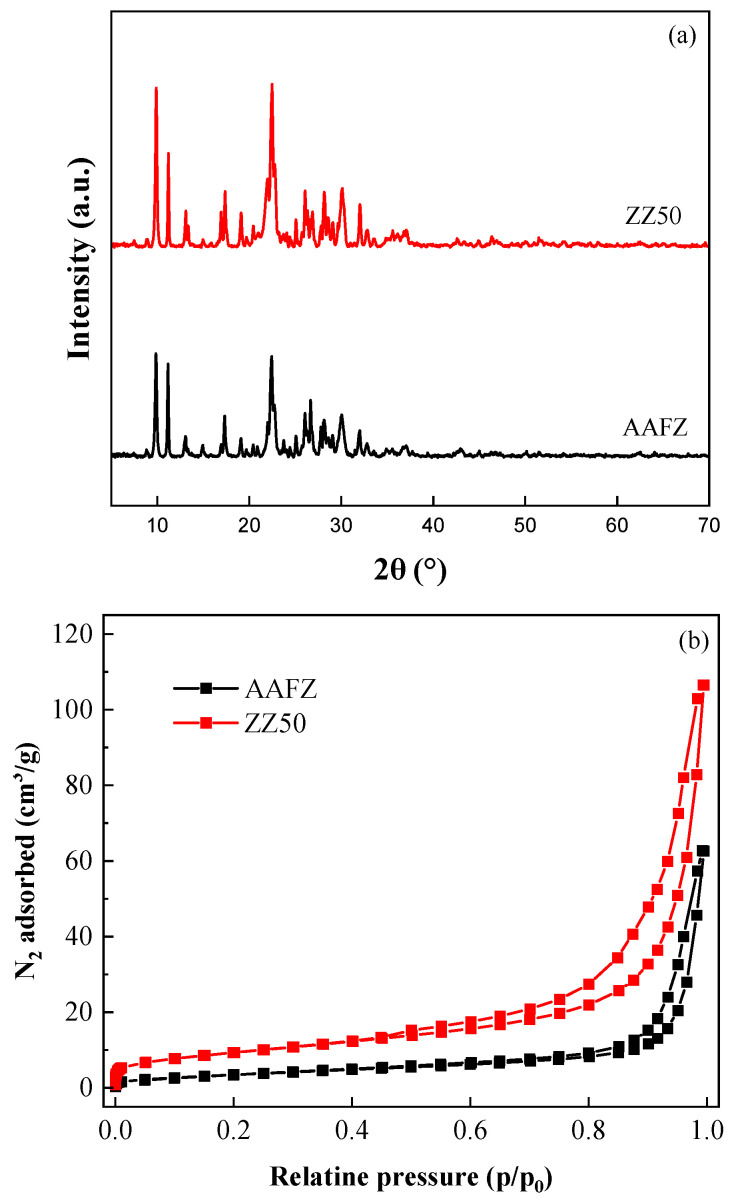

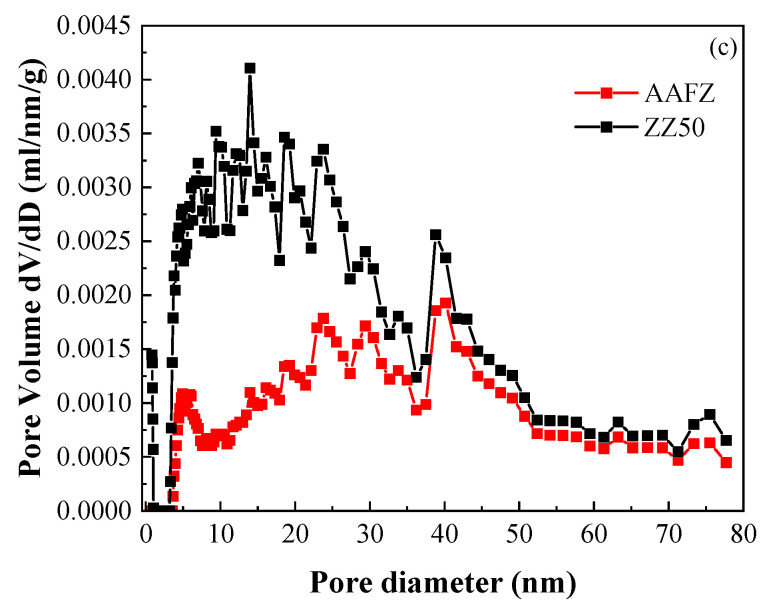
XRD analysis (**a**), N2 adsorption–desorption isotherms (**b**), and pore diameter (**c**) of ZZ50, and AAFZ.

XRD analysis was conducted to examine the crystalline structure of the samples, as illustrated in Figure 1a. Notably, both materials display characteristic reflections consistent with the crystalline phase of clinoptilolite, a defining feature of natural zeolite. Minor minerals such as feldspars, clays, and mica are also identifiable, contributing to the overall composition. Of particular note is that the AAFZ sample reveals the presence of an amorphous N(K)-A-S-H phase. This observation is deduced from changes in the intensity ratio of primary clinoptilolite reflections (2theta 9.9° and 11.2°) corresponding to the [020] and [200] planes [18]. These alterations in intensity ratio are attributed to increased electron density within the crystal plane, resulting from incorporating Na and K cations [19].

Furthermore, specific peak intensities exhibit reduction compared to ZZ50, likely due to partial disruption of the crystal structure induced by alkaline activation or overlap with the amorphous N(K)-A-S-H phase [16]. The identified structural modifications highlight the hybrid composition of AAFZ samples, indicating a dynamic interplay of chemical reactions and phase transformations during synthesis. This coexistence of an amorphous phase with the crystalline clinoptilolite structure suggests intricate structural changes with potential implications for their adsorption properties and effectiveness in environmental remediation applications. As crystallinity significantly influences the adsorption capacity by governing the pore structure and reactivity of the material, a ZZ50 structure with reduced crystallinity offers enhanced accessibility to internal functional groups and improved diffusion properties, thus making it more favorable for adsorption purposes in environmental remediation applications [20].

**Figure 2:**
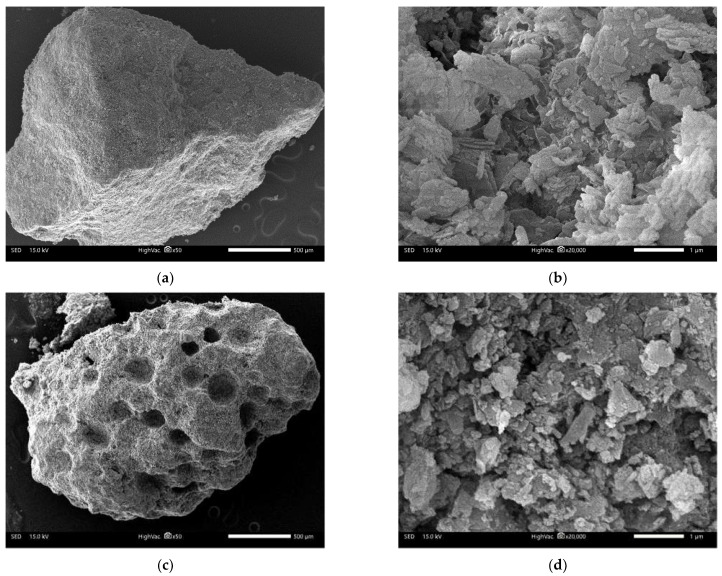
SEM images of ZZ50 (**a**,**b**), and AAFZ (**c**,**d**).

The surface areas and pore structures of both samples were analyzed using nitrogen adsorption experiments. These experiments revealed significant differences in the surface characteristics and pore structures of ZZ50 and AAFZ. As illustrated in the isotherms of Figure 1b, both samples exhibited type IV isotherms and H3 hysteresis loops, indicating the formation of mesoporous structures [21]. According to Table 2, ZZ50 displayed a BET surface area and total pore volume of 34.30 m^2^/g and 0.16 cm^3^/g, respectively. In contrast, AAFZ exhibited lower BET surface area (13.6 m^2^/g) and total pore volume (0.09 cm^3^/g). The reduction in surface area observed in the AAFZ sample compared to ZZ50 can be attributed to several factors, primarily related to the alkali activation process. During this process, the alkaline activator fills the mesopores, leading to pore blockage and ultimately reducing the surface area. Notably, the average pore diameter of AAFZ (17.26 nm) was 1.29 times larger than that of ZZ50 (13.38 nm), indicating differences in pore size distribution (Figure 2c). This variation could be attributed to the foaming of the alkaline-activated mixture with gaseous oxygen generated from the decomposition of hydrogen peroxide in a strongly alkaline medium. This foaming process could result in the formation of larger pores, thereby reducing the overall surface area. Additionally, the significantly lower mesopore volume in AAFZ (0.059 cm^3^/g) compared to ZZ50 (0.123 cm^3^/g) suggests disparities in mesopore development, further contributing to the reduction in surface area. The absence of micropore volumes in both samples suggests a lack of microporous structures, emphasizing the prevalence of mesopores in their pore architecture.

**Figure 3:**
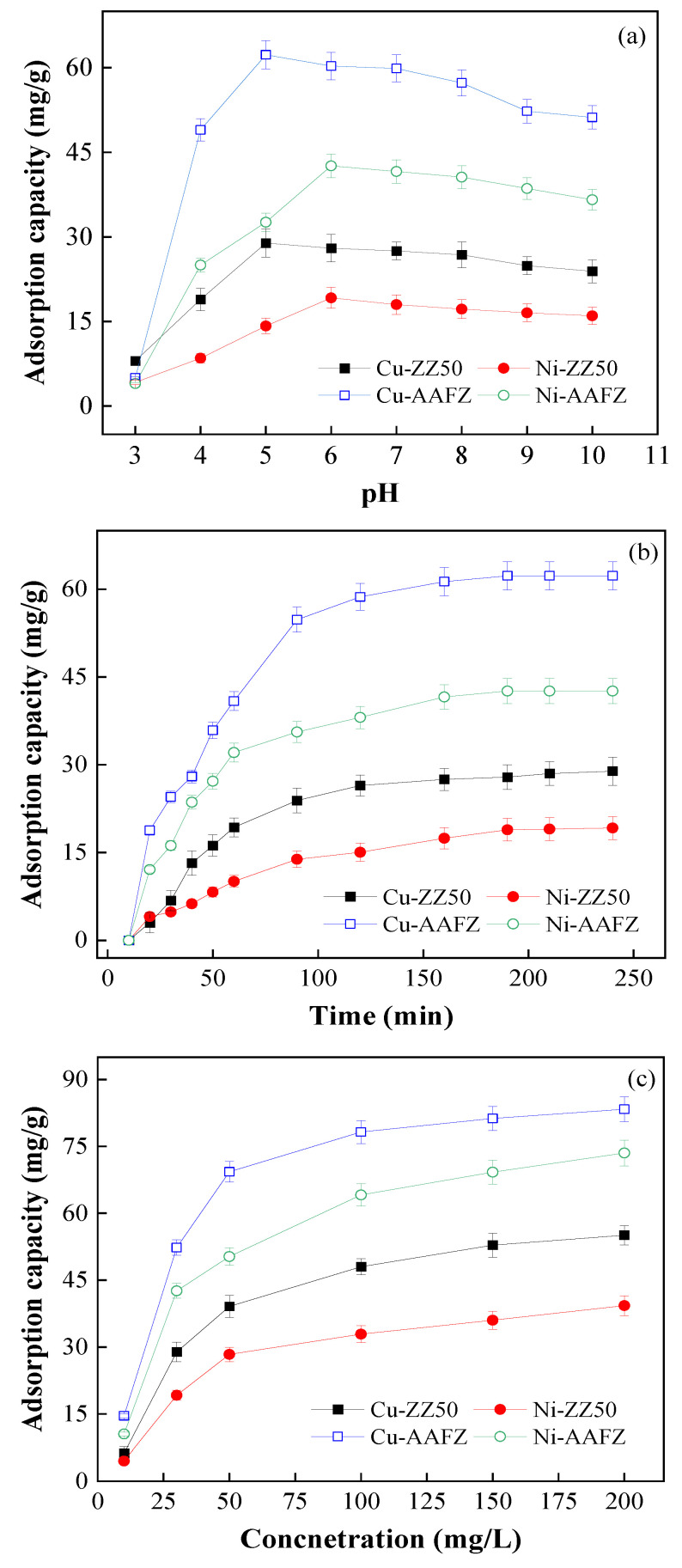
Effect of pH (**a**), time (**b**), and metal ion concentrations (**c**) on Cu(II) and Ni(II) adsorption capacity of ZZ50 and AAFZ.

SEM images of the ZZ50 and AAFZ samples are presented in Figure 2, providing insights into the surface characteristics of these materials. The images reveal a diverse structural arrangement adorned with pores of varying sizes and morphologies. The unmodified ZZ50 sample exhibits a distinct pore distribution characterized by pronounced structural heterogeneity and compacted layer formations, resulting in fewer visible pores (Figure 2a,b). In contrast, the modified sample displays a porous framework with more evenly distributed pores. These images illustrate discernible distinctions between the unmodified ZZ50 sample and the foamed AAFZ adsorbent (Figure 2c,d). Notably, within the AAFZ material, the SEM imagery clearly captures the presence of bubbles generated through the foaming additive’s influence during the zeolite foam synthesis (Figure 3).

### 2.2. Effect of pH

The pH of a solution plays a pivotal role in influencing the adsorption process. Figure 3a illustrates the impact of solution pH on Cu(II) and Ni(II) adsorption capacities on ZZ50 and AAFZ. Consistently, adsorption capacity has a noticeable enhancement with increasing pH levels. Elevated pH levels significantly increased the adsorption of heavy metals onto the adsorbents. This observed trend is in accordance with previous literature [22,23]. At low pH values, both adsorbents exhibit minimal equilibrium adsorption due to a higher concentration of free hydrogen ions (H^+^) in the solution, which compete with heavy metal ions for the same adsorption sites.

Consequently, functional groups become protonated, elevating the cationic charge on the adsorbent surface. This leads to electrostatic repulsion between the functional groups of the adsorbent and heavy metal cations in the solution [24]. As pH increases, the number of free H+ ions decreases, resulting in the deprotonation of adsorption sites. This process diminishes electrostatic repulsion, facilitating increased removal of metal ions from the solution. At pH 5.0, the adsorption capacity of both adsorbents for Cu(II) rapidly reaches its maximum. ZZ50 and AAFZ achieve maximum Cu(II) adsorption capacities of approximately 28.91 mg/g and 62.30 mg/g, respectively, at pH 5. For Ni(II), maximum adsorption capacities of ZZ50 and AAFZ occur at pH 6, reaching 19.20 mg/g and 42.61 mg/g, respectively. However, further increases in pH result in a slight decrease in adsorption capacities. Excessive pH increases may precipitate metal ions due to the formation of hydroxides such as Ni(OH)_2_ [25] and Cu(OH)_2_ [26]. Therefore, pH levels higher than 5 for Cu(II) and 6 for Ni(II) were avoided.

### 2.3. Effect of Time

To determine the optimal time required to achieve adsorption equilibrium, investigations were conducted on the adsorption kinetics of Cu(II) and Ni(II) using specific adsorbents over varying time intervals. Changes in the concentrations of the adsorbates in solutions were meticulously monitored across different periods, and the resulting data are depicted in Figure 3b. The findings delineate the adsorption process into two distinct phases. The initial phase, lasting approximately 60 min, displayed rapid adsorption. During this stage, the adsorption capacity of ZZ50 and AAFZ notably increased, with values reaching 19.30 mg/g and 40.90 mg/g for Cu(II) removal, and 10.71 mg/g and 32.20 mg/g for Ni(II) removal, respectively. This escalated adsorption rate can be ascribed to the facile access to pores within the adsorbents and the abundant availability of adsorptive active sites on their surfaces during the early stages [27].

Subsequently, the process transitioned into a more gradual phase characterized by a slower increase in the extent of adsorbate ion removal compared to the initial phase. Over time, as the adsorption sites on the adsorbent surface became increasingly occupied, the adsorption rate decelerated, eventually attaining a pseudo-equilibrium state. This equilibrium state was established around the 120-min mark for both adsorbents. This behavior arises from the saturation of functional groups on the adsorbent surface due to the binding process between the adsorbate ions and these groups [28]. At the 2-h mark, the maximum adsorption capacity of ZZ50 and AAFZ for Cu(II) and Ni(II) removal was determined to be 26.45, 58.70, 15.61, and 38.10 mg/g, respectively.

### 2.4. Effect of Adsorbate Concentration

The impact of varying initial concentrations of adsorbates, ranging from 10 to 200 mg/L, on the adsorption efficiency of ZZ50 and AAFZ was thoroughly examined. The corresponding experimental data are presented in Figure 3c. It is apparent that in both adsorption systems, the adsorption capacity of the adsorbents demonstrates an increasing trend with the escalation in initial adsorbate concentration. Specifically, in the Cu(II) adsorption system, the adsorption capacity of ZZ50 and AAFZ elevates from 6.21 and 14.60 mg/g to 55.08 and 83.32 mg/g, respectively, as the initial adsorbate concentration progresses from 10 to 200 mg/L. This pattern is also evident in the Ni(II) adsorption system, where the adsorption capacity of ZZ50 and AAFZ reaches 39.30 and 73.52 mg/g, respectively, as the initial Ni(II) concentration reaches 200 mg/L. Notably, the higher initial concentration significantly contributes to the observed increase in adsorption. This is because higher initial concentrations foster more efficient mass transfer dynamics, overcoming barriers that impede the transfer of adsorbate from the solution to the solid phase. This phenomenon underscores the notion that a higher initial adsorbate concentration generates stronger driving forces, compelling the adsorbate ions to infiltrate the pores of the adsorbents, thereby enhancing adsorption [29]. Additionally, an elevated concentration fosters greater interaction between the adsorbate ions and the adsorbents, thereby expediting adsorption kinetics [30].

### 2.5. Kinetic Study

**Figure 4:**
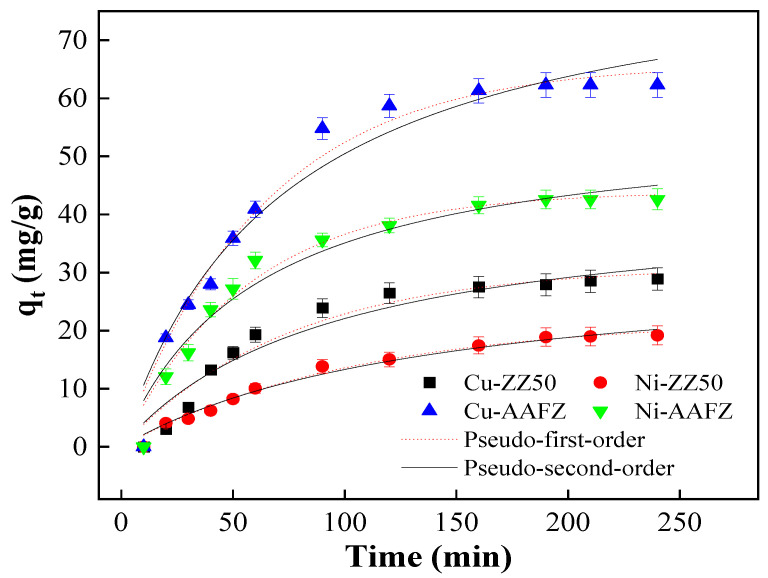
The kinetic models of Cu(II) and Ni(II) adsorption onto ZZ50 and AAFZ.

Figure 4 depicts Cu(II) and Ni(II) adsorption kinetics onto the ZZ50 and AAFZ adsorbents. The findings illustrate heterogeneous adsorption processes characterized by an initial rapid adsorption rate followed by a subsequent slower rate. Both Cu(II) and Ni(II) exhibit swift adsorption onto ZZ50 and AAFZ, reaching equilibrium within one hour. Utilizing kinetic models aids in understanding the adsorption mechanism governing the interaction between the metal ions and the adsorbents. To achieve this, the experimental data were fitted using pseudo-first-order (Equation (1)) and pseudo-second-order (Equation (2)) models, as described in equations [31]:

(1) $$\text{Pseudo-first-order}\;\mathrm{model}:\;\log\left(q_{e}-q\right)=\log\left(q_{e}\right)-\frac{-K_{1}t}{2.303}$$

(2) $$\text{Pseudo-second-order}\;\mathrm{model}:\;\frac{t}{q_{t}}=\frac{1}{K_{2}q_{e}^{2}}+\frac{t}{q_{e}}$$

$q_{t}$ = Adsorption capacities at time t (mg/g);

$q_{e}$ = Adsorption capacities at equilibrium (mg/g);

K_1_ = The rate constants of pseudo-first-order (1/min);

K_2_ = The rate constants of pseudo-second-order (g/mg/min).

The adsorption kinetics of Cu(II) and Ni(II) on ZZ50 and AAFZ are depicted in Figure 4, showing variations in contact times. Remarkably, the kinetic profiles for the heavy metal ions exhibit similar trends. Both Cu(II) and Ni(II) demonstrate a rapid increase in adsorption within the initial hour, consistent with the observed patterns.

The parameters calculated from the kinetic models are summarized in Table 3. Upon analysis, it becomes evident that the adsorption kinetic data for Cu(II) and Ni(II) on ZZ50 and AAFZ align well with the pseudo-second-order model. This observation is supported by notably high correlation coefficients (R^2^), indicating the model’s strong fit to the data. Additionally, lower Chi-square (χ^2^) values further confirm the model’s compatibility with the experimental data. Furthermore, the calculated adsorption capacities derived from the pseudo-second-order model closely approximate the experimental values. These findings collectively suggest that the adsorption mechanism governing the interaction between Cu(II) and Ni(II) and the ZZ50 and AAFZ adsorbents likely involves chemisorption, a phenomenon characterized by the exchange or sharing of electrons between the adsorbate and the adsorbent [32].

### 2.6. Isotherm Study

Adsorption isotherms serve as valuable tools for understanding the phenomena at the adsorption interface, shedding light on the physicochemical behavior resulting from the interaction between the adsorbent surface and metal ions. Furthermore, they allow for exploring the maximum adsorption capacities of adsorbents [33]. In the scope of this study, the Langmuir and Freundlich isotherm models (Equations (3) and (4)) were utilized to examine the adsorption isotherms of Cu(II) and Ni(II) on ZZ50 and AAFZ.

(3) Langmuir equation: qe=qmCe1K1+Ce

(4) Freundlich equation: qe=K2Ce1n

K_1_ = Langmuir constant (L/mg)

K_2_ = Freundlich constant (mg/g)/(mg/L)1/n

$C_{e}$ = The equilibrium concentration of the adsorbate (mg/L)

$q_{m}$ = The maximum adsorption capacity (mg/g)

1/n = The adsorption intensity.

**Figure 5:**
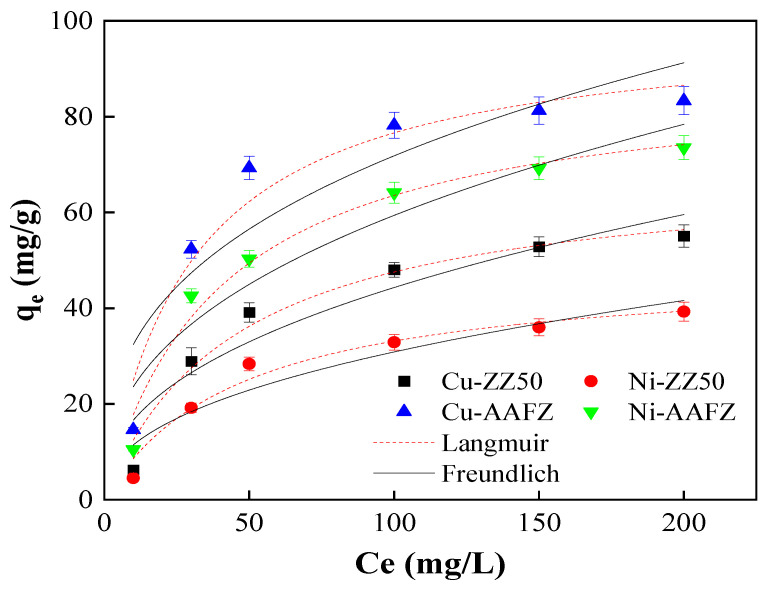
The isotherm models of Cu(II) and Ni(II) adsorption onto ZZ50 and AAFZ.

Figure 5 illustrates the fitting curves, with corresponding parameters detailed in Table 4. The Langmuir and Freundlich plots, depicting the adsorption of heavy metal ions onto ZZ50 and AAFZ adsorbents, are presented in Figure 5. The isotherm curves displayed a gradual slope within the studied range, attributed to the driving force arising from the concentration gradient of metal ions in the system. It is observable that an increase in the metal ion concentrations in both adsorption systems led to enhanced adsorption capacities of ZZ50 and AAFZ for Cu(II) and Ni(II) removal.

Table 4 indicates that the Langmuir model exhibits significantly higher R^2^ and lower χ^2^ values than the Freundlich model. This suggests that the isotherm data fits better with the Langmuir model than the Freundlich model in both adsorption systems. As the Langmuir equation presupposes monolayer adsorption, the good-fitting results imply the potential monolayer adsorption of Cu(II) and Ni(II) on the adsorbents [34]. Additionally, according to the Langmuir isotherm model, the maximum adsorption capacities of Cu(II) on ZZ50 and AAFZ were found to be 69.28 and 99.54 mg/g, respectively. Similarly, the maximum adsorption capacities of Ni(II) on ZZ50 and AAFZ were determined to be 48.53 and 88.99 mg/g, respectively. The RL values, ranging between 0 and 1, indicate favorable adsorption of the metal ions on the adsorbents [35]. Notably, the adsorption capacity of AAFZ for both adsorbate ions surpasses that of ZZ50 considerably. This difference can mainly be attributed to modified adsorption properties of AAFZ, such as larger pore volume (1.29 times), higher Si/Al ratio (1.15 times), and lower crystallinity compared to ZZ50. The Si/Al molar ratio influences the adsorption performance of adsorbents for Cu and Ni ions. AAFZ, with a higher Si/Al ratio compared to ZZ50, exhibits superior adsorption capacity, likely due to increased surface acidity and active sites for ion exchange. This higher Si/Al ratio also contributes to a more stable adsorbent structure, enhancing resistance to leaching and overall adsorption performance. Additionally, crystallinity affects the adsorption capacity by controlling the accessibility of internal functional groups and diffusion properties, allowing better contact with the cations in aqueous solution [20].

Furthermore, it was observed that the trend in the adsorption capacities of heavy metal ions follows the order Cu(II) > Ni(II) in both adsorption systems, indicating a greater affinity of Cu(II) for adsorption compared to Ni(II). The disparity in adsorption behavior between Cu(II) and Ni(II) ions may be attributed to their differing physicochemical factors, including ionic radius, electronegativity, molecular weight, and hydration energy, which are known to influence metal ion adsorption [36,37]. Since Cu(II) has a larger ionic radius and molecular weight, it suggests stronger interactions with the adsorbent. Additionally, the smaller radius of Ni(II) (0.69 Å) compared to Cu(II) (0.73 Å) makes nickel ions easier to hydrate, thus forming a larger water layer on the surface. Consequently, Ni(II) ions are more mobile in bulk solution and would have a lesser tendency to adsorb on the adsorbents [37,38].

Table 5 presents the maximum adsorption capacities of ZZ50 and AAFZ alongside literature values for other adsorbents used in removing Cu(II) and Ni(II) ions. The adsorbents utilized in this study exhibit markedly superior adsorption performance. The findings highlight their high capacities, suggesting significant potential for metal ion removal from aqueous solutions. However, specific adsorbents may be more effective, possibly due to differences in experimental conditions or adsorbent characteristics. ZZ50 and AAFZ demonstrate satisfactory adsorption capacities for reducing Cu(II) and Ni(II) ions.

### 2.7. Regeneration

The examination of an adsorbent’s potential for reusability is paramount for assessing its capacity to regain adsorption efficiency after repeated use and for evaluating its applicability for large-scale operations [49]. Regeneration processes offer the opportunity to diminish the demand for new adsorbents, facilitate resource retrieval, reduce secondary waste generation, and ultimately lower overall operational costs [50]. According to Vakili et al. [51], using acidic eluents has been demonstrated as an effective method for desorbing heavy metals from saturated adsorbents.

**Figure 6:**
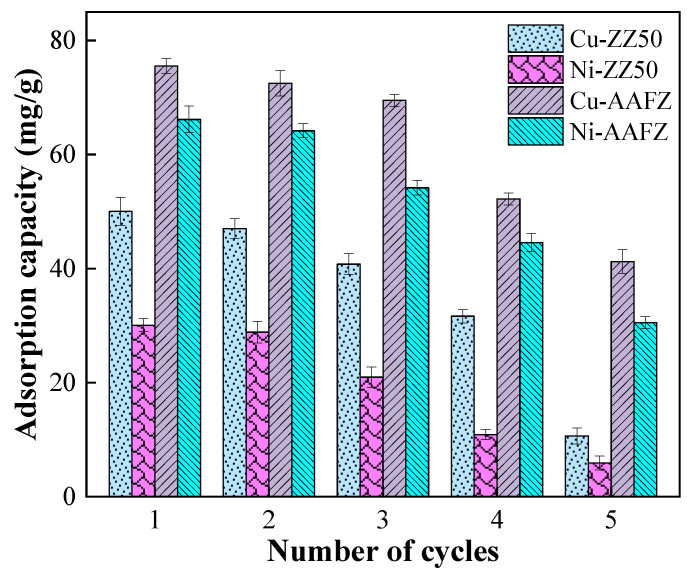
Regeneration of Cu(II) and Ni(II) saturated ZZ50 and AAFZ.

Figure 6 illustrates the reusability of the ZZ50 and AAFZ adsorbents after undergoing five regeneration cycles employing a 0.1 mol/L HCl solution. The findings presented in Figure 6 indicate that both adsorbents maintain substantial adsorption capacities throughout the initial two regeneration cycles and remain relatively stable. However, following the subsequent two cycles, the adsorption capacities for Cu(II) and Ni(II) of AAFZ decrease from 75.52 and 66.16 mg/g to 72.49 and 64.17 mg/g, respectively, representing a loss of less than 3%. This minor reduction could be attributed to the formation of robust complexes between ions and adsorption sites, leading to incomplete desorption of metal ions.

As the number of regeneration cycles increases, the adsorption capacity of AAFZ gradually diminishes, reaching 41.22 and 30.51 mg/g for Cu(II) and Ni(II), respectively, by the fifth cycle. This decline may be linked to the saturation and depletion of available adsorption sites on the adsorbents [52].

## 3. Material and Methods

### 3.1. Materials

For this study, high-quality analytical reagents were obtained from reputable suppliers. Specifically, 30% hydrogen peroxide (H_2_O_2_), magnesium oxide (MgO), and potassium hydroxide (KOH) were sourced from Lach-Ner, s.r.o. (Neratovice, Czech Republic). Sodium silicate with a silicate modulus of 3.22 was provided by Labar s.r.o (Ústí nad Labem, Czech Republic). Zeolite ZEOCEM 50 (ZZ50) in powdered form with a particle size fraction of 560–850 μm was procured from Zeocem (Bystré, Slovakia). Additionally, metal nitrates, including copper nitrate trihydrate (Cu(NO_3_)_2_·3H_2_O) and nickel(II) nitrate hexahydrate (Ni(NO_3_)_2_·6H_2_O), were obtained from Acros Organics B.V.B.A., (Geel, Belgium) and Penta, s.r.o., (Prague, Czech Republic), respectively, to facilitate the experimental procedures.

### 3.2. Preparation of Adsorbents

The adsorbent materials employed in this study were prepared using the same method detailed in our earlier publication [16]. The AAFZ specimens were prepared through a carefully designed procedure involving blending powdered ZZ50 with specific chemical agents. This process aimed to achieve desired characteristics conducive to efficient adsorption performance. The alkaline activator, a crucial component in the synthesis, comprises a solution consisting of water, sodium silicate, and a concentrated KOH solution. The precise formulation of the activator involved the meticulous mixing of water (151 g) with sodium silicate (388 g) and a 40 wt% KOH solution (166 g). The prepared alkaline activator was then introduced into a blend containing ZZ50 (780 g) and MgO (20 g), followed by thorough homogenization through stirring. To remove entrapped air, the mixture underwent vibration using a specialized vibrating pad for 20 s.

Subsequently, the prepared mixture was combined with a foaming agent at a concentration of 0.125% by weight, followed by adding an H_2_O_2_ solution (30%) at a specific concentration. After adding the foaming agent, the mixture underwent uniform mixing and was once again subjected to vibration before being poured into plastic molds. This allowed the material to set and foam over a predetermined period of one hour.

The resulting solidified AAFZ blocks were then subjected to a controlled heating process at 50 °C for 48 h to activate their adsorption properties. After activation, the consolidated AAFZ blocks were carefully placed within airtight plastic bags and underwent a 30-day aging process under ambient laboratory conditions to ensure optimal performance.

**Figure 7:**
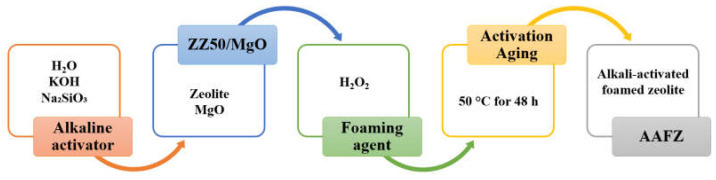
Scheme of AAFZ synthesis.

Following the aging process, the obtained AAFZ blocks were crushed using a laboratory jaw crusher and further separated into distinct particle size fractions using a laboratory sieve shaker. Specifically, the fraction ranging between 560–850 μm was selected for subsequent adsorption assessments, ensuring consistency and accuracy in the experimental procedures. Figure 7 depicts a graphical representation of the synthesis procedure utilized for preparing the AAFZ.

### 3.3. Characterization

The textural properties of ZZ50 and AAFZ were evaluated using the AutoPore IV 9510 system by Micromeritics Instrument Corporation, headquartered in Norcross, GA, USA, for Brunauer–Emmett–Teller (BET) analysis. Crystallinity was analyzed via X-ray diffraction (XRD) utilizing a D8 Advance ECO powder diffractometer manufactured in Germany. Elemental compositions were determined using X-ray fluorescence (XRF) analysis conducted with an S8 Tiger instrument by Bruker AXS GmbH in Karlsruhe, Germany. Additionally, the surface morphologies and chemical compositions of ZZ50 and AAFZ were examined using scanning electron microscopy (SEM) coupled with an Energy-dispersive X-ray spectroscopy detector (EDX) on a JSM-7500F instrument manufactured by JEOL Ltd., headquartered in Tokyo, Japan.

### 3.4. Adsorption and Desorption Experiments

Batch adsorption experiments were conducted to assess the efficacy of the developed adsorbents in removing Cu(II) and Ni(II) ions [53]. These experiments involved agitation on a rotary shaker at 180 rpm with a mixture of 100 mL of adsorbate solutions and 50 mg of adsorbent at room temperature (25 °C). Various adsorption parameters were systematically investigated to determine optimal conditions and enhance adsorption efficiency. These parameters included the pH of the adsorbate solutions (ranging from 2 to 8), contact time (ranging from 10 to 240 min), and adsorbate concentration (ranging from 10 to 200 mg/L).

Kinetic experiments were carried out to evaluate the rate of metal adsorption, varying the contact time from 10 min to 4 h. Adsorbents were immersed in 100 mL of adsorbate solutions (200 mg/L) and agitated at 180 rpm and 25 °C. Adsorption isotherm studies involved introducing adsorbents into 100 mL solutions containing Cu(II) and Ni(II) ions at 10 to 200 mg/L concentrations. Subsequently, samples were extracted from the solution, and the adsorbate concentration was determined after filtration using a 0.22 μm nylon filter. Following adsorption, the remaining concentrations of Cu(II) and Ni(II) were quantified using an Inductively Coupled Plasma Optical Emission Spectrometer (ICP-OES) provided by Agilent Technologies, Inc., headquartered in Santa Clara, CA, USA. The adsorption capacity of the prepared adsorbents was calculated using the following equations:

(5) $$q_{e}=\frac{\left(C_{0}-C_{eq}\right)\times V}{m}$$

where:

- $q_{e}$ represents the amount of metal adsorbed (mg/g);
- $C_{0}$ denotes the initial metal concentration in the solution (mg/L);
- $C_{eq}$ signifies the equilibrium concentration of the metal in the solution (mg/L);
- V stands for the volume of the solution (L);
- m corresponds to the dry weight of the adsorbents (g).

In a series of batch experiments, we examined the reusability of ZZ50 and AAFZ by subjecting them to repeated adsorption and desorption cycles. Initially, we introduced 100 mL solutions containing Cu(II) and Ni(II) ions at a concentration of 100 mg/L to the adsorbents. The mixture was then agitated at 180 rpm for four hours. Afterward, we carefully separated the exhausted adsorbents and thoroughly rinsed them with deionized water to remove any remaining metal contaminants. Next, we treated the saturated adsorbents with 100 mL of 0.1 mol/L HCl and maintained agitation at 180 rpm overnight. We then filtered the solution, extensively washed the adsorbents, and dried them overnight at 50 °C to ensure complete regeneration [31]. The regenerated adsorbents were subsequently used for additional adsorption cycles to evaluate their reusability comprehensively. We conducted all experiments in triplicate to ensure the reliability of our results.

## 4. Conclusions

This research focuses on the development, characterization, and applicability of zeolite-based adsorbents synthesized via alkali activation and foam formation utilizing natural zeolite. The main objective was effectively removing Cu(II) and Ni(II) ions from aqueous solutions. The prepared adsorbents were comprehensively analyzed using SEM, XRF, XRD, and BET techniques to unveil their properties. Chemical composition analysis post-alkaline activation revealed significant alterations. The presence of Na-silicate in the alkaline activator utilized for the AAFZ sample resulted in an enhanced Si/Al molar ratio (1.15 times). XRD analysis verified the presence of crystalline phases alongside an amorphous phase in ZZ50 and AAFZ samples, while AAZF exhibited lower crystallinity. Adsorption behavior was assessed through isotherm and kinetic studies. The pseudo-second-order kinetic and Langmuir isotherm models displayed strong agreement with the adsorption data. Additionally, the maximum monolayer adsorption capacities for Cu(II) and Ni(II) ions were determined as 69.28 and 99.54 mg/g for ZZ50 and AAFZ, and 48.53 and 88.99 mg/g for Ni(II), respectively. AAFZ consistently outperformed ZZ50 owing to its superior pore size, lower crystallinity, and higher Si/Al molar ratio. As indicated by fitting to the pseudo-second-order kinetic model, the adsorption mechanism suggests chemisorption. This study highlights the potential of zeolite as a platform for developing cost-effective adsorbents tailored for removing toxic ions from aqueous environments. The synthesized adsorbents demonstrated promising capabilities in addressing heavy metal-contaminated water, particularly AAFZ. These results contribute to the repertoire of environmentally conscious and economically feasible solutions for mitigating the risks associated with heavy metal contamination in water sources.

## Figures and Tables

**Table 1:** The results of the XRF analysis conducted on the ZZ50, and AAFZ.

Adsorbents	Oxide Parameter (Wt.%)								
	SiO_2_	Al_2_O_3_	TiO_2_	MgO	Na_2_O	CaO	Fe_2_O_3_	K_2_O	Si/Al
ZZ50	75.30	12.60	0.23	0.78	0.30	4.28	1.91	4.26	5.07
AAFZ	68.80	10.00	0.16	3.19	5.12	2.72	1.19	8.63	5.84

**Table 2:** Textural properties of ZZ50, and AAFZ.

Items	ZZ50	AAFZ
BET surface area (m^2^/g)	34.3	13.6
Total pore volume (cm^3^/g)	0.16	0.09
Average pore diameter (nm)	13.38	17.26
Mesopore volume (cm^3^/g)	0.123	0.059
Micropore volume (cm^3^/g)	0	0

**Table 3:** Parameters of the pseudo-first-order and pseudo-second-order models for the adsorption of Cu(II) and Ni(II) onto the adsorbents.

Kinetic Parameters	Cu(II)		Ni(II)	
	ZZ50	AAFZ	ZZ50	AAFZ
Pseudo-first order				
$q_{e}$ (mg/g)	28.91	62.30	19.2	42.60
$q_{cal}$ (mg/g)	42.93	86.62	32.14	56.47
$K_{1}$ (1/min)	2.470	1.611	2.201	2.912
χ^2^	7.871	21.53	0.946	10.59
R^2^	0.936	0.960	0.981	0.946
Pseudo-second order				
$q_{e}$ (mg/g)	28.91	62.30	19.2	42.60
$q_{cal}$ (mg/g)	31.11	66.01	22.05	43.98
$K_{2}$ (g/mg/min)	0.013	0.016	0.010	0.018
χ^2^	6.035	13.90	0.720	7.154
R^2^	0.951	0.972	0.986	0.967

**Table 4:** Parameters of the Langmuir and Freundlich isotherm models for Cu(II) and Ni(II) adsorption onto the ZZ50 and AAFZ adsorbents.

Isotherm Parameters	Cu(II)		Ni(II)	
	ZZ50	AAFZ	ZZ50	AAFZ
Langmuir				
$q_{m}$ (mg/g)	69.28	99.54	48.53	88.99
K1 (L/mg)	0.021	0.033	0.022	0.025
χ^2^	12.86	44.62	6.99	18.92
R^2^	0.970	0.948	0.966	0.972
R_L_	0.192	0.131	0.185	0.166
Freundlich				
$K_{2}$ (mg/g)	6.222	14.62	4.244	9.378
n	2.345	2.894	2.321	2.49
χ^2^	46.30	152.6	22.10	70.42
R^2^	0.891	0.824	0.894	0.896

**Table 5:** Comparative analysis of Cu(II) and Ni(II) adsorption across various adsorbents.

Adsorbent	Adsorbate	q_m_ (mg/g)	pH	Time (h)	Ref.
Willow wood (1 mg/L)	Cu(II) (0.05–160 mg/L)	12.2	5.2	0.5	[39]
	Ni(II) (0.05–160 mg/L)	9.8	6.1	0.5	[39]
3-aminopropyl triethoxysilane-microfibrillated cellulose (0.67 mg/L)	Cu(II) (10–300 mg/L)	2.74	5	5	[40]
	Ni(II) (10–300 mg/L)	2.63	5	5	[40]
Polyacrylonitrile/Na-Y-zeolite (1.00 g/L)	Cu(II) (5–100 mg/L)	54.80	4	1.5	[41]
Hydroxylamine-polyacrylonitrile micro/nanofibers (0.1 g/L)	Cu(II) (10–300 mg/L)	105	5.5	8	[36]
	Ni(II) (10–300 mg/L)	104	5.5	6	[36]
4-phenylacetophynone 4-aminobenzoylhydrazone silica gel (5 g/L)	Cu(II) (1–20 mg/L)	0.76	6	2	[42]
	Ni(II) (1–20 mg/L)	0.82	7	2	[42]
SiO_2_-NH-HPBT (1 g/L)	Cu(II) (2–200 mg/L)	20.5	5	3	[32]
	Ni(II) (2–200 mg/L)	23.0	6	5	[32]
FAU zeolite (5 g/L)	Cu(II) (100–500 mg/L)	57.80	-	1	[43]
Zeolite (25 g/L)	Cu(II) (30–1600 mg/L)	114.94	5	2	[44]
SA/ZSM-5 (4 g/L)	Ni(II) (25–100 mg/L)	19.60	6	0.75	[45]
NaP zeolite (2.5 g/L)	Cu(II) (10–250 mg/L)	42.9	5.2	5	[46]
LTA zeolite (2.5 g/L)	Cu(II) (10–250 mg/L)	140.1	5.2	1	[46]
Methacrylate-Na-Y-Zeolite (0.5 g/L)	Cu(II) (5–100 mg/L)	37.10	4.5	1.5	[47]
Zeolite (0.2 g/L)	Cu(II) (10–50 mg/L)	6.74	5	1	[48]
ZZ50 (0.5 g/L)	Cu(II) (10–200 mg/L)	69.28	5	2	Present study
	Ni(II) (10–200 mg/L)	48.53	6	2	
AAFZ (0.5 g/L)	Cu(II) (10–200 mg/L)	99.54	5	2	Present study
	Ni(II) (10–200 mg/L)	88.99	6	2	

## Data Availability

Data are contained within the article.
